# Efficient Neural Decoding Based on Multimodal Training

**DOI:** 10.3390/brainsci14100988

**Published:** 2024-09-28

**Authors:** Yun Wang

**Affiliations:** 1 Institute of Science and Technology for Brain-Inspired Intelligence, Fudan University, Shanghai 200433, China; 19110850009@fudan.edu.cn; 2 Key Laboratory of Computational Neuroscience and Brain-Inspired Intelligence, Fudan University, Ministry of Education, Shanghai 200433, China

**Keywords:** neural decoding, multimodal pre-training, diffusion model, fusion transformer, scene reconstruction

## Abstract

Background/Objectives: Neural decoding methods are often limited by the performance of brain encoders, which map complex brain signals into a latent representation space of perception information. These brain encoders are constrained by the limited amount of paired brain and stimuli data available for training, making it challenging to learn rich neural representations. Methods: To address this limitation, we present a novel multimodal training approach using paired image and functional magnetic resonance imaging (fMRI) data to establish a brain masked autoencoder that learns the interactions between images and brain activities. Subsequently, we employ a diffusion model conditioned on brain data to decode realistic images. Results: Our method achieves high-quality decoding results in semantic contents and low-level visual attributes, outperforming previous methods both qualitatively and quantitatively, while maintaining computational efficiency. Additionally, our method is applied to decode artificial patterns across region of interests (ROIs) to explore their functional properties. We not only validate existing knowledge concerning ROIs but also unveil new insights, such as the synergy between early visual cortex and higher-level scene ROIs, as well as the competition within the higher-level scene ROIs. Conclusions: These findings provide valuable insights for future directions in the field of neural decoding.

## 1. Introduction

Neural decoding, which infers mental representations from brain activities, also known as “mind reading’’, has attracted considerable attention. Neural decoding is not only useful for extracting sensory and motor information but also finds applications in brain–machine interfaces [1]. Furthermore, it holds potential for investigating brain disease states that could impact perception and cognition [2,3,4].

**Figure 1:**
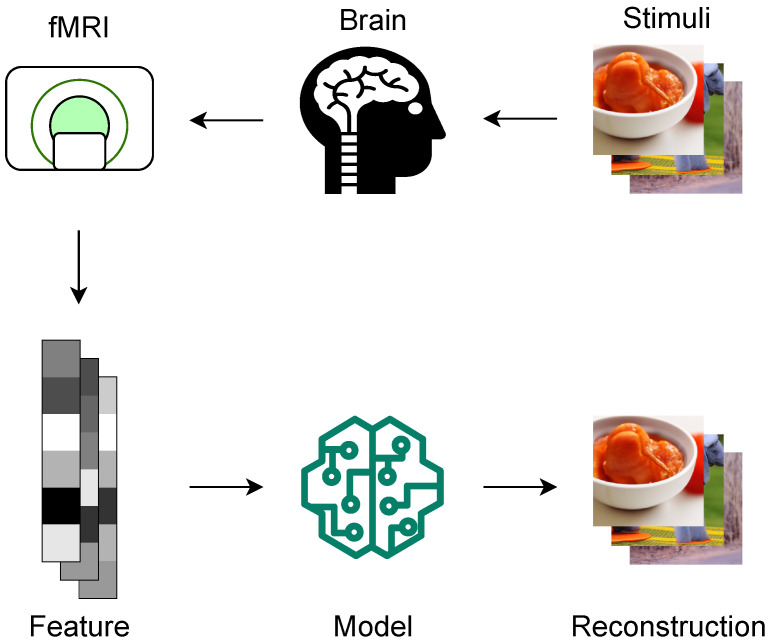
Neural decoding of visual image. Subjects undergo fMRI scanning while viewing visual stimuli. The brain activity corresponding to stimuli is recorded and transformed into features. Computational models reconstruct the original stimuli based on the features.

Previous studies have explored decoding various types of information from brain activities, such as orientation [5], color [6], shape [7], attention [8], category [9], language [10], and reward [11]. Neural decoding of visual image from fMRI has been extensively studied recently. As illustrated in Figure 1, researchers present subjects with various visual stimuli while they undergo fMRI scanning. The brain response to these stimuli is then analyzed to identify patterns of activity that correlate with different types of stimuli. With a computational model, the original stimuli are reconstructed from the observed brain activity, which can be a powerful tool for understanding brain function.

One of the key points for high-quality visual decoding is to learn an fMRI encoder with rich representation of visual information. The main challenge in neural decoding of perceived images is the scarcity of fMRI data paired with images, limiting learned representation of fMRI data. Popular datasets for visual decoding, such as the Generic Object Decoding Dataset (GOD) [12], BOLD5000 [13], and the Natural Scene Dataset (NSD) [14], have fewer than ten thousand fMRI-image pairs for every participant. Therefore, it will limit the performance of decoding models directly trained on these data. Previous research has demonstrated enhanced decoding performance through unimodal pre-training on resting-state fMRI data [15]. However, due to the absence of visual information in resting-state fMRI data, a vast amount of data and model parameters are required for pre-training.

Previous studies on visual decoding have primarily aimed to enhance reconstruction quality, while applying decoding models to uncover novel findings needs further research. Few studies decode images from ROIs to confirm past findings about ROIs [16,17,18]. Therefore, further efforts are needed to reveal new knowledge of the functional properties of brain ROIs via decoding.

To this end, we present a three-stage neural decoding model based on multimodal training on image–fMRI pairs to enable the high-quality decoding of images from few fMRI data. Our contributions are as follows:

- We propose a multimodal masked autoencoder (MAE) [19] jointly trained on paired image–fMRI data to better encourage the fMRI encoder to learn information from image modality, enabling effective and efficient encoding of fMRI data.
- By combining the representational power of a multimodal trained model with the generative capabilities of latent diffusion model (LDM), we enhance the quality of decoding, resulting in more realistic images when compared to earlier state-of-the-art methods.
- Additionally, we utilize the decoding model to explore the representation of each ROI. Through visualization, we not only validate previous studies but also derive new insights into the functional roles of scene-related ROIs.

## 2. Related Work

Conventional decoding approaches have mapped brain data to image features extracted by deep neural networks (DNNs) and employed generative models like generative adversarial networks (GANs) and variational autoencoders (VAEs) to reconstruct perceived or imagined images [12,20,21]. While these methods captured image features accurately, they often struggled to produce visually clear images [22] and Ozcelik et al. [17], Mozafari et al. [23] introduced linear regression models to extract latent factors from fMRI data, which were then used to fine-tune BigGANs. To overcome the limitations of training on small paired fMRI–image datasets, self-supervised learning techniques were introduced, allowing for the integration of unlabeled image and brain data during training [24,25].

Diffusion models have since outperformed GANs in image generation tasks [26,27]. These models operate by gradually corrupting input images with noise in forward processes, and then recovering the original image by estimating the noise at each step in reverse processes. While diffusion models working directly in image space can be computationally expensive, latent diffusion models (LDMs) encode images into a more efficient latent space, enabling the generation of high-resolution images with lower computational costs. Chen et al. [15] advanced this approach by pre-training a masked autoencoder (MAE) on large-scale resting-state fMRI data to encode brain signals and subsequently training an LDM conditioned on these encoded signals to produce high-quality images. However, training a brain data encoder on massive unimodal fMRI data alone is both resource-intensive and could benefit from improvements offered by multimodal approaches [28,29].

Recent studies have explored mapping fMRI data to CLIP space for decoding using diffusion models. For example, Ozcelik and VanRullen [18], Takagi and Nishimoto [30] proposed linear mappings that require training large, high-dimensional regression models with numerous parameters. Similarly, Scotti et al. [31] employed a large-scale multilayer perceptron (MLP) to encode fMRI data and used a diffusion prior in the CLIP’s image embedding space. These models also rely on numerous parameters, making them prone to overfitting, particularly on smaller datasets.

## 3. Materials and Methods

### 3.1. Overview

**Figure 2:**
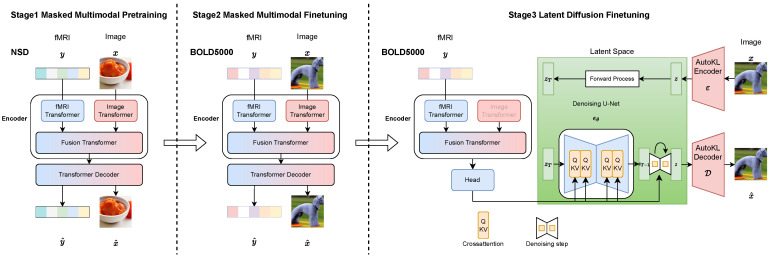
Proposed pipeline of decoding with multimodal training.

Our decoding pipeline consists of three stages, as illustrated in Figure 2. First, a multimodal image–fMRI MAE is trained to reconstruct images and brain signals utilizing a large-scale fMRI dataset, e.g., the Natural Scene Dataset. Subsequently, the pre-trained MAE undergoes fine-tuning on the target decoding dataset. Finally the fine-tuned fMRI encoder will be jointly fine-tuned with a latent diffusion model (LDM).

Similar to multimodal pre-training with paired image and text data [32], multimodal pre-training of paired image and brain data encourages cross-modal information modeling between brain activities and image information, which is beneficial for the downstream visual decoding task.

### 3.2. Multimodal Masked Autoencoder

Masked autoencoders utilize encoders operating on a small proportion of patches and decoders reconstructing original data from the latent representation and mask tokens to accelerate training of large models and improve performance. Here, we followed Georgescu et al. [33] to use a mid-fusion multimodal MAE with a shared decoder transformer, and the standard transformer architecture was used [34,35]. The inputs for the decoder are derived from encoding the unmasked tokens and reinserting the mask tokens, denoted as $z_{f}\in R^{n_{f}\times d}$ and $z_{v}\in R^{n_{v}\times d}$. Here, $z_{f}$ and $z_{v}$ represent the fMRI and image modalities, respectively. Likewise, $n_{f}$ and $n_{v}$ indicate the total number of fMRI and image tokens. The joint reconstruction objective $L_{rec}$ is designed to reconstruct the original fMRI and image inputs that correspond to the mask tokens in $z_{f}$ and $z_{v}$, which is decomposed into an fMRI modality objective $L_{f}$ and an image modality objective $L_{v}$. Given mask tokens and original signals ${\hat{x}}_{f}$ and ${\hat{x}}_{v}$, the objectives are computed as follows:

(1) $$\begin{array}{cc} \; & L_{f}\left(z_{f},{\hat{x}}_{f}\right)=\frac{1}{\alpha_{f}n_{f}}\sum_{i\in M_{f}}{∥Decode\left(z_{f,i}\right)-{\hat{x}}_{f,i}∥}^{2}\\ \; & L_{v}\left(z_{v},{\hat{x}}_{v}\right)=\frac{1}{\alpha_{v}n_{v}}\sum_{i\in M_{v}}{∥Decode\left(z_{v,i}\right)-{\hat{x}}_{v,i}∥}^{2}\\ \; & L_{rec}=0.5\times L_{f}+0.5\times L_{v}, \end{array}$$

where $\alpha_{f}$ and $\alpha_{v}$ are the mask ratios, and $M_{f}$ and $M_{v}$ are the sets of mask token indices. For each modality, the encoder transformer had 12 layers, an embedding dimension of 768, 3072 MLP size, and 12 attention heads. The last 2 layers of the encoders were shared between modalities. The patch embedding of the fMRI encoder utilized a 1-dimensional convolution layer with kernel and stride equal to a patch size of 16. The image and fMRI mask ratio were both set to 0.75. The decoder transformer had 4 layers, an embedding dimension of 512, 2048 MLP size, and 8 attention heads.

### 3.3. Latent Diffusion Models

Diffusion models [26,27,36,37] encompass forward processes that add noise to images and reverse processes that remove noise to recover images. Latent diffusion models employ autoencoders to transform between the image space and the latent space and perform a diffusion process in the latent space. For an image *x*, the LDM encoder E encodes *x* into a latent embedding z=E(x), on which the diffusion and denoising processes are applied. The decoder D then reconstructs the image from this latent representation. The LDM uses a time-conditional U-Net architecture [38] as the backbone for the denoising network $\epsilon_{\theta }$, which is augmented with a cross-attention mechanism [35] to condition the model on various input modalities. In this work, an fMRI encoder $\tau_{\theta }$ is employed to project the fMRI data *y* into an intermediate embedding that conditions the denoising process. Given an image–fMRI pair (x,y), the conditional LDM is trained using the following objective:

(2) $$L:=E_{E(x),y,\epsilon ∼N(0,1),t}\left[{∥\epsilon -\epsilon_{\theta }\left(z_{t},t,\tau_{\theta }\left(y\right)\right)∥}_{2}^{2}\right],$$

where *t* is the time step in the forward diffusion process, ϵ represents Gaussian noise, $z_{t}$ is the latent representation at time step *t* with added noise, and the denoising network $\epsilon_{\theta }$ predicts the noise at each step. In this study, we followed [15] to encode each fMRI into the same shape as the CLIP text embeddings of 1×77×768 with linear mapping. A denoising diffusion implicit model sampler with 50 denoising steps was used for sampling [39]. Classifier-free guidance was used to improve generation quality. Sampling was perform using the linear combination of the conditional estimates $\epsilon_{\theta }\left(z_{t},t,\tau_{\theta }\left(y\right)\right)$ and unconditional estimates $\epsilon_{\theta }\left(z_{t},t\right)$

(3) $${\hat{\epsilon }}_{\theta }\left(z_{t},t,\tau_{\theta }\left(y\right)\right)=\left(1+w\right)\epsilon_{\theta }\left(z_{t},t,\tau_{\theta }\left(y\right)\right)-w\epsilon_{\theta }\left(z_{t},t\right),$$

where *w* is the scale controlling guidance strength. As a text-to-image latent diffusion model was used, unconditional estimates were obtained by encoding empty text. Notably, classifier-free guidance is expected to induce a reduction in diversity, aligning with the objective of neural decoding.

### 3.4. Decoding Training Stages

#### 3.4.1. Stage 1: Masked Pre-Training

For pre-training, the Natural Scene Dataset (NSD) [14] was utilized. NSD scanned 8 subjects viewing images from the COCO dataset [40] with high-resolution 7 Telsa fMRI. Each image trial was presented for 3 s in a continuous recognition task. The fMRI response–betas extracted by the general linear model of each image trial on fsaverage [41] space were mapped to the fsLR 32k [42] space using Connectome Workbench V1.5 [43]. V1, V2, V3, and V4 ROIs were selected from the Human Connectome Project (HCP) multimodal parcellation [44], resulting in a total of 4183 vertices. The publicly released sessions of NSD were employed, and trial betas from the same image were averaged. Unique images of all subjects were used for training, while shared images were used for validation, resulting in approximately 67k samples for training and 6k for validation. To align with the patch size, 4183 fMRI vertices were flattened to 1d and padded to 4192. The pre-training hyperparameters were set as follows: batch size of 512, learning rate of 2 × 10^−4^, 300 epochs, Adam [45] optimizer with betas (0.9, 0.98), weight decay of 0.01, and a cosine annealing learning scheduler [46]. Data augmentation involved random cropping and resizing images to 224×224, as well as random augmentation.

#### 3.4.2. Stage 2: Masked Fine-Tuning

We chose BOLD5000 [13] as our target decoding dataset due to its extensive range of images sourced from ImageNet [47], MS COCO [40], and various scenes. And we present results mainly for subject CSI1. Pairs of image and fMRI from BOLD5000 were used for fine-tuning on the checkpoints from stage 1. The ROIs selected for this stage were early visual cortex (EVC), lateral occipital complex (LOC), occipital place area (OPA), parahippocampal place area (PPA), and retrosplenial complex (RSC). The number of voxels was padded from 1685 to 1696. Testing was performed on 113 out of 4916 images, while the remaining images were used for training. The fine-tuning hyperparameters included a batch size of 64, learning rate of $1\times 10^{-4}$, 100 epochs, Adam optimizer with betas (0.9, 0.98), weight decay of 0.01, and a cosine annealing learning scheduler. Mean squared error (MSE) loss was employed for reconstruction, and data augmentation was consistent with stage 1.

#### 3.4.3. Stage 3: Fine-Tuning Latent Diffusion Model

The third stage involved joint fine-tuning the MAE encoder from stage 2 with a latent diffusion model on BOLD5000. Only the cross-attention heads of the LDM were trained, following the suggestion of Chen et al. [15]. A linear map was utilized to map from the MAE output to the CLIP text embedding shape. The stable diffusion v1.5 checkpoint was used for the LDM. Similar to MinD-Vis [15], the fine-tuning hyperparameters were set as follows: batch size of 32, learning rate of $5.3\times 10^{-5}$, 500 epochs, Adam optimizer with betas (0.9, 0.999), and weight decay of 0.01. Data augmentation involved random cropping and resizing images to 256×256 and 20% random drop of fMRI voxels.

### 3.5. Evaluation Metrics

The evaluation metrics in this study are as follows: Structural Similarity Index (SSIM) [48], which measures the similarity between two images by considering changes in structural information, luminance, and contrast; two-way comparisons between reconstructed and ground truth images at the second convolutional layer of AlexNet, evaluating the preservation of critical visual features; two-way comparisons at the final pooling layer of Inception V3 [49], assessing the similarity in high-level abstract features; two-way comparisons at the output layer of the CLIP vision model [32], comparing the output embeddings that encapsulate both visual and semantic information; and 50-way classification accuracy [25], which quantifies the ability of the model to maintain distinct class-specific features in the reconstructed images. The low-level metrics include SSIM and AlexNet(2), while the high-level metrics include Inception, CLIP, and 50-way classification accuracy.

### 3.6. ROI Analysis

To gain insights into the specific function of brain ROIs, images were decoded from the artifical pattern of ROIs with our method. Some ROIs were activated and some were deactivated. The response amplitude of the activated ROIs was set to 1, while the deactivated ROIs were set to −1. The ROIs of EVC, LOC, OPA, PPA, and RSC were included in this analysis.

## 4. Results

### 4.1. Qualitative Results

**Figure 3:**
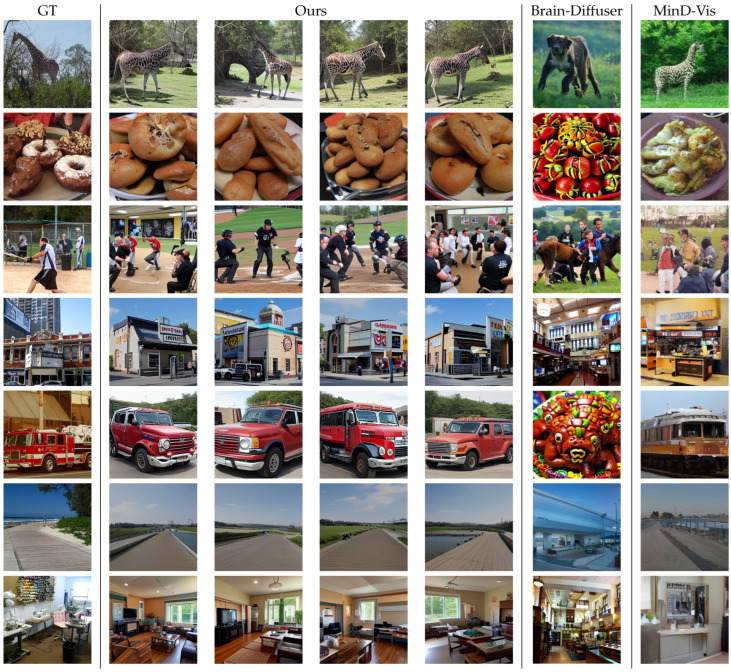
Qualitative results.

We present reconstruction examples from our model in Figure 3. For each test image, four random samples were shown for our method with classifier-free guidance of 1.5. Additionally, we generated one example for each image using MinD-Vis and Brain-Diffuser. Our method can accurately decode semantic contents and achieve low-level alignment across diverse categories, including humans, animals, food, buildings, objects, indoor scenes, outdoor scenes, etc.

We illustrate the proficiency of our approach through several examples. In the first instance, our model accurately decodes a giraffe in a forest, capturing the background color and texture more closely to the ground truth than MinD-Vis, whereas Brain-Diffuser incorrectly decodes a dog. In the second example, although the fine-grained category is not an exact match, our model decodes a plate of food with colors and textures that are closer to the ground truth, outperforming both MinD-Vis and Brain-Diffuser. The third example involves people playing baseball, which our model accurately decodes, unlike MinD-Vis and Brain-Diffuser, which struggle with identification. In the fourth instance, our method successfully reconstructs a building with exterior text, whereas MinD-Vis and Brain-Diffuser generate incorrect indoor scenes. In the fifth example, our model reconstructs a red vehicle, despite some mismatches in fine-grained details, while MinD-Vis shows significant color and appearance shifts, and Brain-Diffuser generates an incorrect category. The sixth example, an outdoor scene with the sky, road, and horizon, is reconstructed with remarkable fidelity by our model. Finally, in the seventh example, our model decodes an indoor scene featuring furniture and a window with high accuracy.

### 4.2. Quantitative Results

Table 1 presents a quantitative comparison of our method against MinD-Vis and Brain-Diffuser. Our method demonstrates superior performance on both low-level and high-level metrics compared to MinD-Vis, which relies on unimodal pre-training with extensive fMRI data. Moreover, our results exceed those of Brain-Diffuser, which employs a simpler linear mapping technique.

In terms of low-level metrics, our approach significantly outperforms the others in SSIM, achieving a score of 0.433, which indicates a higher structural similarity between the reconstructed images and the ground truth. This is notably better than MinD-Vis (0.319) and Brain-Diffuser (0.193), highlighting the effectiveness of our multimodal pre-training in capturing fine-grained details. In the AlexNet(2) metric, which assesses feature similarity using a pre-trained AlexNet model, our method achieves 0.740, surpassing both MinD-Vis (0.724) and Brain-Diffuser (0.736). It again suggests that our method is better at preserving low-level visual features.

For high-level metrics, our method shows a consistent improvement across all evaluated metrics. Our method achieves an Inception accuracy of 0.738, compared to 0.716 for MinD-Vis and 0.666 for Brain-Diffuser. The CLIP accuracy also shows our method’s superiority with a score of 0.826, whereas MinD-Vis and Brain-Diffuser score 0.806 and 0.803, respectively.

Finally, our method achieves the highest 50-way classification accuracy (0.263), which is a direct indicator of how well the reconstructed images can be correctly classified into one of 50 categories, compared to 0.259 for MinD-Vis and 0.198 for Brain-Diffuser. This further underscores the performance of our approach.

We attribute the effectiveness of our approach to the training process, which involves the joint reconstruction of image and fMRI pairs, enabling the capture of a richer set of low-level and high-level features that are reflected in our qualitative results.

### 4.3. Computational Complexity Analysis

**Figure 4:**
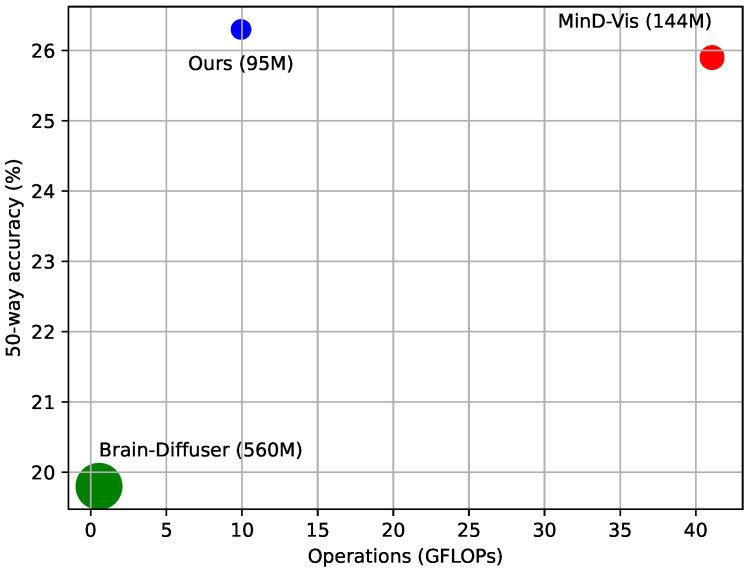
50-way accuracy and computational complexity.

In addition to the qualitative and quantitative evaluation of our approach for decoding, it is crucial to consider the computational complexity of our model in comparison to existing methods. This section provides an analysis of the computational resources required by our model, MinD-Vis, and Brain-Diffuser in terms of floating-point operations (FLOPs) and number of parameters. Figure 4 illustrates the relationship between the computational complexity, measured in GFLOPs, and the 50-way classification accuracy for the three models. The size of the dots corresponds to the number of model parameters. Our model achieves the highest 50-way accuracy with only one quarter the computational complexity of MinD-Vis. This efficiency is a result of our novel multimodal pre-training approach, which leverages paired image and brain data to establish a more efficient brain encoder. Our method can decode high-quality images from fMRI data with less computational overhead, making it a more scalable solution for practical applications. In addition, our proposed model contains 95 million parameters, which is a significant reduction compared to MinD-Vis with 144 million parameters and Brain-Diffuser with 560 million parameters. In summary, our model achieves high decoding accuracy and computational efficiency, making it a strong candidate for practical deployment in neuroscience research and applications.

### 4.4. Ablations

In order to assess the efficacy of pre-training and fine-tuning, we conducted ablations of stagewise training. The evaluation encompassed four experimental settings: (1) training the MAE fMRI encoder and LDM cross-attention heads solely on BOLD5000 at Stage 3 without any pre-training, (2) training on BOLD5000 at Stages 2 and 3, (3) pre-training on NSD at Stage 1 followed by fine-tuning on BOLD5000 at Stage 3, and (4) pre-training on NSD at Stage 1 followed by fine-tuning on BOLD5000 at Stages 2 and 3. Table 2 demonstrates the impact of different stages of training on various evaluation metrics. Additional Stage 1 or Stage 2 outperforms the individual use of Stage 3 across multiple metrics, suggesting the effectiveness of multimodal training. “Stage 1+Stage 2+Stage 3’’ is superior to “Stage 1+Stage 3’’ and “Stage 2+Stage 3’’, indicating the importance of both multimodal pre-training and fine-tuning. Overall, the results emphasize the importance of stagewise training, with the combination of all three stages consistently outperforming other configurations across all metrics.

### 4.5. ROI Analysis

**Figure 5:**
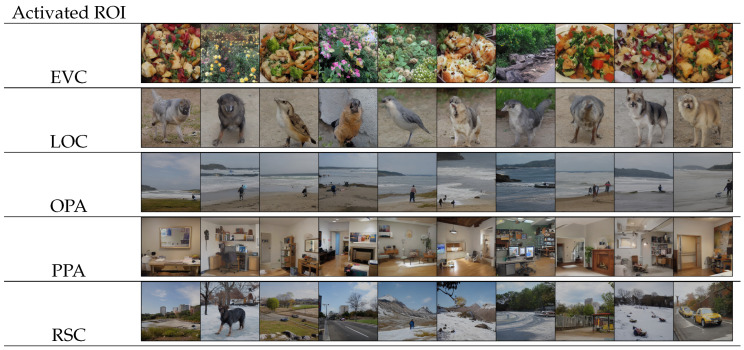
Images derived from synthetic fMRI patterns generated through the activation of one ROI.

Our decoding model could be potentially used to reveal the function of ROIs through decoding. Following Ozcelik and VanRullen [18], the investigation aimed to discern the information represented in ROIs through the utilization of artificial fMRI patterns, thereby illuminating the function of ROIs. The generation of ROI-optimal images involved setting deactivated ROIs to a value of −1 and activated ROIs to 1. To ensure consistency, ten random images were sampled for each synthetic fMRI pattern. The outcomes, as illustrated in Figure 5, mirrored earlier findings in Ozcelik and VanRullen [18] and supported some previous studies. Specifically, activating EVC while inhibiting other ROIs produced textures such as plants and food. Activation of LOC appeared to generate animal representations such as birds and dogs. PPA activation yielded indoor scenes characterized by dense objects. On the other hand, activation of OPA or RSC resulted in distinct outdoor scenes, with OPA producing open scenes featuring water and humans, while RSC led to scenes depicting land, potentially accompanied by objects.

Our findings provide support for prior investigations on the functional characteristics of scene-related ROIs. The activation of LOC has been linked to the representation of object information [50,51,52], as well as scene content [53], which aligns with the observed generation of animal-related representations upon LOC activation. Similarly, OPA has been associated with encoding local elements within scenes [54] and delineating boundaries [55], and thus may explain the emergence of scenes depicting land-water demarcations upon OPA activation. PPA, on the other hand, has been implicated in representing spatial boundary information [53], rectilinear features [56], cardinal orientations [57], and indoor scenes [58], providing a plausible account for the generated indoor scenes. Furthermore, RSC has shown heightened responsiveness to spatial layout information [51] and is involved in the mnemonic aspects of scene processing [59]. This characteristic may account for the presence of inconsistent objects within scenes, as the brain actively processes information in RSC, potentially reflecting the inherent randomness in fMRI data, subsequently captured by the model.

**Figure 6:**
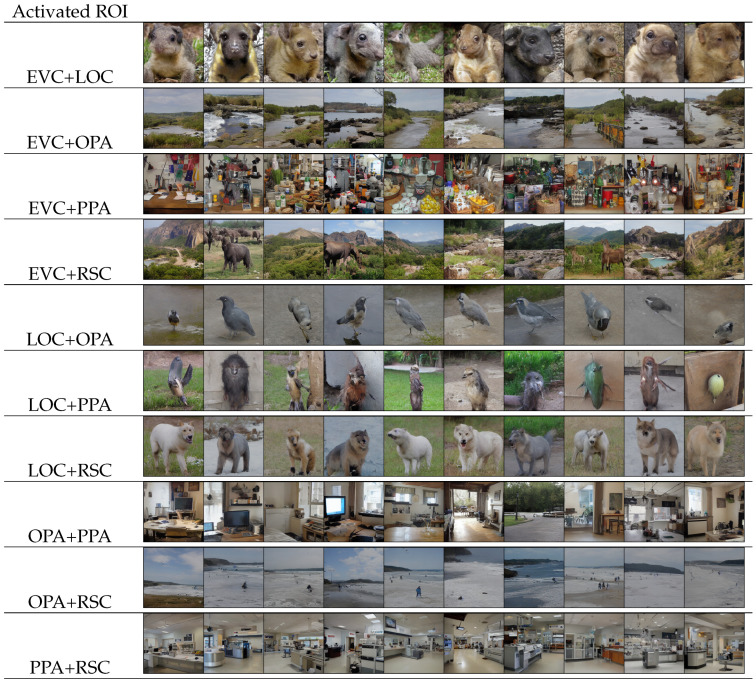
Images derived from synthetic fMRI patterns generated through the activation of two ROIs.

In addition, we conducted experiments involving the coactivation of two ROIs alongside the inhibition of other ROIs, as depicted in Figure 6. The coactivation of two ROIs can be viewed as activating one ROI then varying another ROI from −1 to 1. Intriguingly, EVC and higher cortices exhibited a synergistic effect on the neural representations. Specially, the activation of EVC, in conjunction with higher cortices, resulted in the inclusion of plants within outdoor objects and scenes, while indoor scenes exhibited heightened complexity. The coactivation of EVC and LOC, which are both involved in size perception [60], not only altered the background but also significantly changed object size.

Moreover, the coactivation of higher cortices displayed a competitive relationship between them. Notably, when coactivated with other scene-related ROIs, LOC consistently prevailed, giving rise to the generation of animal representations. During the coactivation of OPA or RSC, PPA took precedence in producing indoor scenes. Additionally, the coactivation of OPA and RSC appeared to yield open scenes characterized by the presence of water and humans, potentially indicative of OPA dominance over RSC. Note that as those fMRI patterns are artificial, the results reflect the knowledge assimilated by the model from the underlying data, which may be affected by distribution imbalance. In light of this, it is imperative to underscore that the aforementioned hypothesis warrants further confirmation to attain scientific validity.

## 5. Discussion

Upon recent advances in decoding images from fMRI, the perceived images of the brain can be decoded with high quality. In this study, we propose multimodal pre-training and fine-tuning of an fMRI encoder on image–fMRI pairs with reconstruction as the optimization target, and jointly fine-tune the fMRI encoder and the cross-attention heads of the LDM. Through qualitative and quantitative comparison with state-of-the-art methods, including MinD-Vis and Brain-Diffuser, our model has shown superior performance in reconstructing both semantic contents and low-level visual attributes from fMRI data. Our study demonstrates the efficacy of multimodal training in improving neural decoding accuracy with fewer data resources and lower computational complexity.

The outcomes of our model indicate a marked improvement in decoding accuracy compared to existing methodologies, affirming our hypothesis that our model can more effectively capture the complexities of brain activity associated with visual processing. One of the key strengths of our model lies in its architecture, which integrates multimodal information. This approach not only enhances the richness of the learned representations but also facilitates a deeper understanding of the relationships between neural activity and visual stimuli.

The application of decoding models to aid computational neuroscience research represents a promising avenue for future investigations. Here, we utilize our decoding model to generate images of artificial fMRI patterns that activate and inhibit ROIs. Our results validate some previous studies on the function of each ROI. Moreover, our decoding model reveals new insights, highlighting the synergistic effect between the EVC and higher scene cortices, along with the existence of competitive processes within higher scene cortices. By leveraging the power of machine learning techniques, we gain deeper insights into brain activity patterns and their underlying cognitive processes.

**Figure A1:**
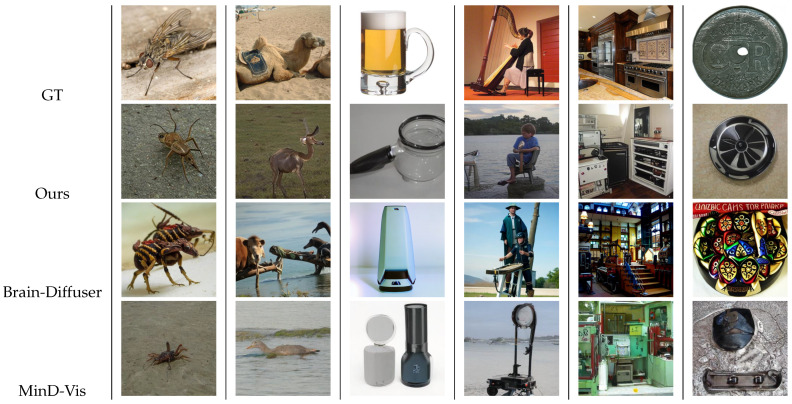
Qualitative results.

Nevertheless, it is crucial to acknowledge the limitations of our study. One limitation is the reliance on a specific dataset for training and validation, which may limit the generalizability of our findings to other datasets or populations. As demonstrated in Figure A1 and Table A1, the results indicate challenges in reconstructing objects from categories not present in the training data. This finding points to the need for improving model decoding performance while generalizing to unseen objects. This issue also underscores the importance of selecting diverse datasets when evaluating reconstruction models in future works. Additionally, the interpretability of our decoding model’s results may be subject to the inherent complexity of neural activity and the assumptions made by the model architecture.

For future work, exploring alternative model architectures or incorporating additional modalities, such as electroencephalogram (EEG)or behavioral data, could enhance the robustness and interpretability of neural decoding models. Investigating the transferability of our findings to clinical applications, such as diagnostic support or neurofeedback interventions, holds promise for translating our research into practical tools for healthcare. Moreover, addressing ethical considerations, such as privacy protection and bias mitigation, will be crucial for the responsible deployment of decoding technologies in real-world settings.

## 6. Conclusions

In conclusion, while our study contributes valuable insights into neural decoding and cognitive neuroscience, further research and validation efforts are necessary to fully realize the potential of decoding models in understanding brain function and behavior. 

## Figures and Tables

**Table 1:** Quantitative analysis. The best value of each metric is in bold. Up arrows indicate the higher the better.

Method	Low-Level		High-Level		
	SSIM↑	AlexNet(2)↑	Inception↑	CLIP↑	50-Way↑
MinD-Vis [15]	0.319±0.146	0.724±0.269	0.716±0.322	0.806±0.227	0.259±0.359
Brain-Diffuser [18]	0.193±0.157	0.736±0.308	0.666±0.310	0.803±0.231	0.198±0.302
Ours	0.433±0.138	0.740±0.278	0.738±0.308	0.826±0.211	0.263±0.347

**Table 2:** Ablation studies of stagewise training. The best value of each metric is in bold. Up arrows indicate the higher the better.

Method	Low-Level		High-Level		
	SSIM↑	AlexNet(2)↑	Inception↑	CLIP↑	50-Way↑
Stage 3	0.287	0.529	0.563	0.582	0.054
Stage 2+Stage 3	0.289	0.573	0.591	0.650	0.090
Stage 1+Stage 3	0.281	0.579	0.588	0.695	0.106
Stage 1+Stage 2+Stage 3	**0.433**	**0.740**	**0.738**	**0.826**	**0.263**

## Data Availability

The data used in this research are publicly available at [13,14].

## Appendix A. Results on Additional Dataset

To further assess the generalization of our model, we conducted experiments using the Deeprecon Dataset, similar to the approach in MinD-Vis. This dataset includes 1200 training images and 50 test images, with no overlap in categories, providing a rigorous test of model generalization. We compare our method with MinD-Vis and Brain-Diffuser quantitatively in Table A1 and qualitatively in Figure A1.

While all methods demonstrated lower reconstruction accuracy on this dataset compared to BOLD5000, no single method consistently outperformed the others quantitatively. These results suggest that decoding unseen objects may require models with more robust generalization capabilities.

**Table A1:** Quantitative analysis. The best value of each metric is in bold. Up arrows indicate the higher the better.

Method	Low-Level		High-Level		
	SSIM↑	AlexNet(2)↑	Inception↑	CLIP↑	50-Way↑
MinD-Vis [15]	0.316±0.160	0.709±0.267	0.691±0.277	0.711±0.307	0.234±0.336
Brain-Diffuser [18]	0.327±0.175	0.863±0.179	0.646±0.334	0.782±0.250	0.100±0.186
Ours	0.334±0.146	0.683±0.278	0.664±0.327	0.721±0.287	0.121±0.255
